# Determinants of childhood diarrhea among underfive children in Benishangul Gumuz Regional State, North West Ethiopia

**DOI:** 10.1186/1471-2431-14-102

**Published:** 2014-04-14

**Authors:** Thomas Sinmegn Mihrete, Getahun Asres Alemie, Alemayehu Shimeka Teferra

**Affiliations:** 1 Central statistical agency of Ethiopia, Assossa Branch, Benishangul Gumuz, Ethiopia; 2 Department of Epidemiology and Biostatistics, College of Medicine and Health Sciences, University of Gondar, Gondar, Ethiopia

**Keywords:** Childhood diarrhea, Benishangul Gumuz Region, Environmental, Socio-economic determinants

## Abstract

**Background:**

Diarrhea is second only to pneumonia as the cause of child mortality worldwide. Developing countries particularly in Sub Saharan Africa including Ethiopia have a high burden of this disease. Studies showed that different factors were associated with the occurrence of childhood diarrhea. Therefore, this study was aimed to identify determinant factors of diarrhea in underfive children in Benishangul Gumuz Regional State, western Ethiopia.

**Method:**

Demographic and Health Survey (DHS) data of 2011 was used for this study. The data was extracted from the National DHS data using data extraction tools. A total of 925 under five children were selected. The logistic regression model was employed to examine the determinants of childhood diarrhoea. Both bivariate and multivariate data analysis was performed using SPSS version 16.0.

**Result:**

The results of this study indicated that low level of maternal education [AOR = 1.81, 95% CI (1.12,2.76)], absence of toilet facility [AOR = 3.5, 95% CI (2.4, 5.2)], improper child stool disposal methods [AOR = 2.05, 95%CI (1.36, 3.10)], having more than two under five children [AOR = 1.73, 95% CI (1.03, 2.93)], higher birth order [AOR = 6.1, 95% CI (3.1,12.2)] and the age of children [AOR = 1.9, 95% CI (1.2, 3.6)] were found to be the risk factors for childhood diarrhea after adjusting for other variables. When toilet facility was stratified by maternal education, it showed that children of mothers who had no education were the most vulnerable in the absence of toilet facilities [OR = 9.16, 95% CI (5.79, 14.48)].

**Conclusion:**

Under poor environmental conditions, mothers with primary education and above protected their children against diarrhea better than mothers with no education. Thus, implementing effective educational programs that emphasize environmental health and sanitation practices and encouraging female school enrolment would reduce childhood diarrheal morbidity in the region.

## Background

Diarrhoea is the second gravest killer of underfive children worldwide [1]. Every year, 2.5 billion cases of diarrhoea likely to result in death or other service outcomes occur among underfive children. More than half of these cases occur in Africa and South Asia. Underfive mortality due to diarrhoea is about 1.5 million each year. About 80% of the deaths are still in Africa, including Ethiopia [2].

Studies and reports on child morbidity and mortality in Ethiopia show that diarrhoea is a major public health problem [3,4]. According to the 2010 report of the Ministry of Finance and Economic Development’s (MOFED), 20% of the childhood death in the country was due to diarrhoea. The 2011 Ethiopian Demographic and Health Survey (EDHS) reported that 13% of the children had diarrhoea in the two weeks preceding the survey at the national level [5,6]. Different community based surveys on childhood morbidity and mortality in Ethiopia at different places disclosed three episodes of diarrhoea per child per year [7]. Deaths attributed to diarrhoea were 23 per 1000 live births [4]. Studies conducted in south west and central Ethiopia revealed that the mortality attributed to diarrhoea was 30% and 27%, respectively [4,8].

The Ethiopian Ministry of Health has been struggling to curb the morbidity and mortality of children by formulating and implementing different policies and strategies. In 2003, the Ministry adopted an integrated management of childhood illness (IMCI) approach as a key national strategy. In 2005, the national strategy for child survival which focused on health service extension program was launched. However, the major focuses of these strategies were increasing access to essential basic curative health services to the majority of the population [9,10]. Despite these continuing efforts, the main issue of preventing morbidity by identifying the basic causes of illness remained important in fighting against child mortality.

Many studies agree that child morbidity and mortality are results of interactions among many factors in developing countries. The interactions of behavioural, socioeconomic and environmental factors influence child morbidity [2,11]. Understanding childhood morbidity requires explaining the relations and interactions of these factors.

The Benishangul Gumuz Regional State has the highest underfive mortality rate which is almost twice the country’s average of diarrheal morbidity [6]. Therefore, this study will seek to identify the determinants of diarrhoea among underfive children in the region for proper planning of intervention and prevention programs. The region had the worst and declining child health outcomes in the past decade. This poor performance can be clearly seen in the rise of the under five diarrhoeal morbidity which rose from 21.1% in 2005 to 22.7% and mortality from 157 to 169 per 1000 live births in the 2011 DHS. This is despite the fact that the country is committed to reducing child morbidity and mortality [6,7].

**Figure 1:**
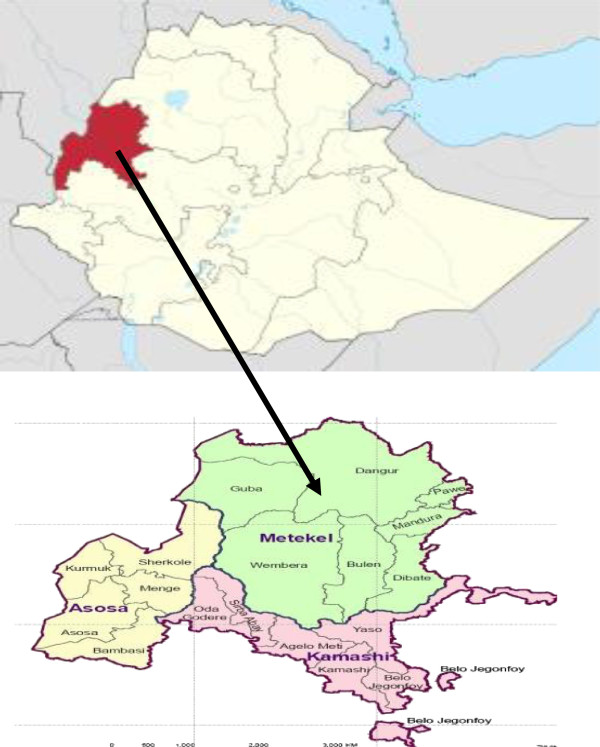
Map and location of Benishangul region in Ethiopia.

Identifying the causes of diarrhea is very crucial for the effective implementation of child health intervention programs for policy formulation and the general assessment of resource requirements and intervention prioritization in the region. Therefore, this study was conducted to identify the risk factors for the occurrence of childhood diarrhea among children aged 0–5 years in Benishangul Gumuz Regional State, northwest Ethiopia (Figure 1).

## Methods

### Study design and period

A secondary data analysis was done on the Ethiopian Demographic and Health Survey 2011, in this population based cross sectional study. The study used the Benishangul Gumuz Regional State DHS data. The region has an estimated area of 51,000 KM^2^. It is located in the western part of Ethiopia. It shares common borders with the Amhara Region in the East, the Republic of Sudan in the northwest, and Oromia Region in the south. The region is divided into three administrative zones and 19 districts. About 75% of the region is classified as low land, which is below 1500 meters above sea level. The altitude ranges from 550 to 2,500 meters above sea level. According to the 2011 CSA estimates, the region has a population of 982, 004. Ninety percent of the population lives in rural areas, indicating the very low level of urbanization. Agriculture is the major economic activity followed by traditional gold mining. The region has two hospitals, 28 health centers, and 339 health posts [12].

### Sample size and sampling techniques

The sample size for this study was 925 under five children. The samples were selected using a two stage stratified cluster sampling technique in the EDHS 2011 for Benishangul Gumuz Region. Initially, the region was stratified into urban and rural clusters. A total of 6 urban and 42 rural clusters were considered, and then all women with 0–5 years of age children were selected in each cluster. Finally, 925 youngest or index children aged 0–5 were taken for the analysis.

### Data collection procedures

Data was extracted from EDHS 2011 children’s data set using the prepared data extraction tool (Additional file 1). The new data set was carefully extracted from EDHS 2011 data regarding environmental and socio-economic determinants of childhood diarrhea among underfive children.

### Data processing and analysis

Extracted data were checked for completeness, coded, and entered into SPSS version 16.0. Binary and multiple logistic regressions were used to assess the association of various determinant factors of childhood diarrhea. The results were presented in the form of tables, figures, and summary statistics. The strength of association of determinant factors with the outcome variable was assessed using the odds ratio with a 95% confidence interval. In a multiple logistic regression analysis all variables that were found significant at p-value of 0.25 and 95% CI in the bi-variate analysis were entered into the model and a backward step wise method was used [13]. Variables which were significant at p-value 0.05 level and 95% CI were considered to be the determinant factors of childhood diarrhea.

Ethical clearance was obtained from the Institute of Public Health Institutional Review Board, the University of Gondar. Official permission was also secured from the Central Statistical Agency (CSA) to use the DHS data set for this study.

## Results

### Socio-economic characteristics

A total of 925 underfive children were included in the study with a response rate of 99.9%. The majority of the children, 847 (91.6%), were from rural areas while the rest, 78 (8.4%), from urban areas of the region. The mean age of their mothers was 28.4 (±6), 60% of whom were below the age of 29. More than half of the mothers, 522 (56.4%) were Muslims followed by 211 (23.1%), Orthodox Christians and 144 (15.8%) protestants. Nearly half of the mothers (48.9%) were not working; 26.2% were farmers, and 27% had non-agricultural occupation. The mean family size of the study population was 5.9 (±2.288) persons. More than half of the households (50.1%) had a family size of 6 or more persons, and the rest had 5 or less persons. More than 80% of the households had 2 or less under five children in the family. The remaining 20% had three or more under five children. The majority of the households (52%) were in the poor category of wealth status, 20.2% in the medium, and 27.5% in the high wealth category [Table 1].

**Table 1:** Socioeconomic and demographic characteristics of respondents in Benishangul in Gumuz region, 2013

**Characteristics**	**Frequency**	**Percent**
**Maternal Education**		
No education	541	58.5
Primary or above	384	39.5
**Occupation of mother**		
Not working	448	48.9
Working	468	51.1
**Place of residence**		
Urban	78	8.4
Rural	847	91.6
**Household size**		
5 or less	462	49.9
6 and above	463	50.1
**No of under five in the HH**		
2 or less	756	81.7
3 and above	169	13.3
**Wealth status**		
Poor	484	52.3
Medium	187	20.2
Rich	254	27.5
**Religion of the mother**		
Orthodox	211	23.1
Muslim	522	56.4
Protestant	146	15.8
Others^†^	11	1.2
**Ethnicity of the mother**		
Amhara	225	24.3
Berta	270	29.2
Gumuz	196	21.2
Oromo	133	14.4
Others*	91	10.9
**Age of Mother**		
15-24	734	79.4
25-34	179	19.4
35-49	12	1.3
**Father’s education**		
No education	509	55.2
Primary or above	412	44.8

^**†**^Traditional belief *Shinasha, Agew.

### Environmental characteristics

Of the total 925 households, 876 (94.7%), had floors made of dirt and only 49 (5.3%) households had non-dirt floor material. Households which had no toilet facility were 396 (42.8%). Regarding disposal of children’s stool, 55% of the households disposed children’s’ stool in an improper manner. Two hundred ninety-eight (32.2%) of the households had unimproved source of water [Table 2].

**Table 2:** Environmental conditions of the study participants in Benishangul Gumuz Region in 2013

**Characteristics**	**Frequency**	**Percent**
**Source of drinking water**		
Improved	627	67.8
Not improved	298	32.2
**Type of toilet facility**		
Pit or flash toilet	529	57.2
No facility	396	42.8
**Main Floor material**		
Dirt	876	94.7
Non dirt	49	5.3
**Child stool disposal**		
Safe	410	44.6
Not safe	510	55.4

### Child demographic characteristics

Out of 925 children, 459 (49.6%) were male and the rest 50.4% female. Of all the under five children, 235 (25.5%), were underweight at birth. The prevalence of diarrhea in the previous two weeks preceding the survey was 22.1% [Table 3].

**Table 3:** Demographic and health characteristics of children in Benishangul Gumuz region in 2013

**Characteristics**	**Frequency**	**Percentage**
**Birth order**		
1	208	22.5
2-3	284	30.7
4-5	236	25.5
6+	197	21.3
**Low birth weight**		
Yes	235	25.5
No	688	74.5
**Age of child in months**		
<6	105	11.7
6-11	89	9.9
12-23	168	18.6
24 and above	539	59.8
**Sex of child**		
Male	459	49.6
Female	466	50.4
**Presence of diarrhea**		
Yes	204	22.1
No	721	77.9

### Determinants of childhood diarrhea

#### Socio-economic determinants

In the bi-variate analysis, only maternal and paternal education, and maternal occupation showed significant association with childhood diarrheal morbidity. Maternal education was found to have a strong association with childhood diarrhea. Children of none educated mothers were about two times more likely to have diarrhea when compared to children of mothers who had primary education and above [COR: 2.27, 95% CI (1.17, 3.20)]. Similarly, mothers’ occupation had a significant association with childhood diarrhea. Children of mothers who had work were about two times more likely to have diarrhea compared to children of mothers who were not working [COR: 1.76, 95% CI (1.28, 2.43)]. Paternal education was also found to be significantly associated with diarrheal morbidity.

### Environmental determinants

Household environmental variables and their relation with childhood diarrhea were assessed on the bi-variate analysis. All variables showed a significant association with childhood diarrhea except household floor material. Children in households where their main drinking water source was not improved were two times more likely to have diarrhea than children from households that had improved water sources [OR: 2.22, 95% CI(1.62, 3.06)].

Similarly, the analysis showed that there was a difference in the likelihood of diarrhea by the type of toilet facility. Children from those households who had no toilet facility had about six times more likelihood to have diarrhea than children from households who had toilet facility [OR: 6.74, 95% CI (4.70, 9.67)]. The analysis showed about a 60% reduction of childhood diarrhea in households who disposed the stool of children in a safe way than those children from households who disposed stool in an unsafe manner [OR: 0.38, 95% CI (0.27, 0.53)].

### Child demographic determinants

In this bi-variate analysis, only the age of a child and birth order were found to be significantly associated with childhood diarrheal morbidity. The risk of diarrheal morbidity was higher at age categories of 6–11 months [OR: 2.02, 95% CI (1.06, 3.83)] and 12–23 months [OR: 2.21, 95% CI (1.25, 3.88)] and lower on the age of 24 months and above compared to 0–5 months of age. Higher birth order of the child had a significant risk for diarrheal morbidity. Being the second or third child had about three times more likelihood to have diarrhea compared to being the first child [OR: 2.82, 95% CI (1.56, 5.08)]. Furthermore, being the sixth child or more had about 6 times more chance of having diarrhea compared to the first child [OR: 5.91, 95% CI (3.28, 10.66)].

### Multivariable analysis

In the bi-variate analysis, unadjusted OR results showed a significant variation in the effects of socio-economic variables as well as varying degrees of the control variables on the risk of childhood diarrhea. However, any possible confounding factors were not controlled at this level; assessing the independent effects of the covariates was difficult. The back ward step wise regression technique was used to assess the relative effect of independent variables on the response variable. To rule out the excess of variables and unstable estimates in the final model, only variables that reached a p-value of less than 0.25 in the bi-variate analysis were included in the multivariable analysis [14]. In the multivariable analysis Hosmer and Lemeshow goodness of fit test was performed [13], and it was found that the model was good (p-value = 0.20). Finally maternal education, maternal occupational status, number of under five children, type of toilet facility, child stool disposal, age of child and birth order had significant associations with diarrheal morbidity. According to the result of the multivariate analysis, children of mothers who had no education had about two times higher odds of diarrhea than children whose mothers’ had primary and above education [AOR: 1.81, 95% CI (1.12, 2.76)]. The risk of diarrhea in children whose mothers were farmers was the same in children whose mothers were not working, but the risk was doubled in children whose mothers had non-agricultural occupation compared to children whose mothers were not working [AOR: 2.11, 95% CI (1.34, 3.32)]. When the number of under five children in the households was two or less, the risk of diarrhea decreased by about 42% compared to households who had more than three underfive children [AOR: 0.58, 95% CI (0.34, 0.98)]. The type of toilet facility had a significant association with diarrheal morbidity. Children from households who had no toilet facility had six times more risk for having diarrhea than children from families who had toilet facilities [AOR: 5.90, 95% CI (3.93, 8.86)]. Also safe disposal of child stool was found protective of childhood diarrheal morbidity by about 50% [AOR: 0.49, 95% CI (0.34 0.78)]. The risk of diarrhea peaked at the age of 12–23 months [AOR: 1.94, 95% CI (1.43, 3.78)], and higher ages and birth orders had a significant association with childhood diarrheal morbidity [Table 4].

**Table 4:** Multivariate analysis of determinant factors of childhood diarrhea in Benishangul Gumuz Region, Ethiopia in 2013

**Characteristics**	**Diarrhea**		**Crude OR (95% CI)**	**Adjusted OR (95% CI)**	**P-value**
	**Yes**	**No**			
**Maternal education**					
No education	149	392	2.27 (1.62,3.20)	1.81 (1.12,2.76)*	0.027
Primary and above	55	329	1.00	1.00	
**Occupation of mother**					
Not working	76	372	1.00	1.00	0.035
Working	124	344	1.76 (1.28, 2.43)	1.62 (1.10, 2.38)	
**Father’s education**					
No education	100	409	1.381 (1.011,1.86)*		
Primary and above	104	308	1.00		
**No. of under five in the house**					0.043
3+	172	584	1.00	1.00	
2 or less	32	137	0.79 (0.52,1.21)	0.58 (0.34, 0.98)*	
**Source of drinking water**					
Improved	109	518	1.00		
Not improved	95	203	2.22 (1.62, 3.06)*	-	
**Type of toilet facility**					
Pit or flush toilet	47	482	1.00	1.00	0.000
No facility	157	239	6.74 (4.70,9.67)*	5.90 (3.93, 8.86)*	
**Main floor material**					
Dirt	197	679	1.74 (0.77,3.94)	-	
Non dirt	7	42	1.00		
**Child stool disposal**					0.003
Safe	148	361	0.38 (0.268,0.532)*	0.49 (0.34,0.78)*	
Not safe	55	355	1.00	1.00	
**Age of child in months**					
< 6	22	83	1.000	1.00	0.052
6-11	31	58	2.02 (1.06,3.83)*	1.41 (0.65,3.07)	
12-23	62	106	2.21 (1.25,3.88)*	1.94 (1.43,3.78)*	
24 and above	87	141	1.10 (0.61,1.97)	0.76 (0.32,1.47)	
**Birth order**					
1	16	192	1.00	1.00	0.001
2-3	54	230	2.82 (1.56,5.08)*	3.70 (1.87,7.31)*	
4-5	69	167	4.96 (2.77,8.87)*	6.68 (3.35,13.30)*	
6+	65	132	5.91 (3.28,10.66)*	6.72 (3.33,13.54)*	

*Statistically significant at p-value < 0.05.

The type of toilet facility was stratified by maternal education and revealed that it varied with the level of education of mothers on childhood diarrhea. In the absence of toilet facility, children from mothers who had no education had a very high risk of developing diarrhea [OR: 9.16, 95% CI (5.79, 14.48)] than children from mothers who had primary and above education [OR: 3.73, 95% CI (2.03, 6.83)]. Therefore there was an interaction between toilet facility and maternal education [Table 5].

**Table 5:** Stratified analysis of type of toilet facility by mother’s education on the effect of diarrhea in Benishangul Gumuz region, 2013

**Mother’s education**	**Type of toilet facility**	**Had diarrhea**		**OR 95% CI**
		**Yes**	**No**	
	Pit or flush	47	482	Crude OR 6.74 (4.70, 9.67)*
	No facility	157	239	
**No education**	Pit or flush	29	270	9.16 (5.79, 14.48)*
	No facility	120	122	
**Primary & above**	Pit or flush	18	212	3.73 (2.03, 6.83)*
	No facility	37	117	

*Statistically significant at p-value < 0.05.

## Discussion

This study has revealed the important determinants of childhood diarrhea among under five children. Out of environmental variables, the type of toilet facility and stool disposal were found to be significantly associated with childhood diarrhea.

Children from households with no toilet facility were about six times more at risk of diarrhea than children living in households with toilet facility; this finding is consistent with the findings in Egypt, Ethiopia, and Lesotho [8,15-18]. The way the child’s stool is disposed may significantly affect the occurrence of childhood diarrhea. Children from households who disposed stool properly had more than 50% reduction of the risk of childhood diarrhea. These findings are consistent with the study done in Tigray Region of Ethiopia on childhood morbidity [8]. The type of toilet facility and stool disposal schemes might shade light on the notions of household sanitary conditions and as such on the possibility of the transmission of diarrheal pathogens through fecal contamination [2,19].

Of all the socio-economic variables considered, only maternal education and occupation and the number of underfive children in the household remained significant after controlling child demographic and health, environmental and other socio-economic variables.

The findings of this study regarding maternal education emphasizes that mothers with lower educational status put their children at higher risk for having diarrhea, which is in line with a community-based cross-sectional study in Ethiopia, Zimbabwe, Uganda, India and in a case control study done in Lesotho [15,17,18,20-23]. Mother’s literacy influences hygienic practice, child feeding, weaning and sanitation practices which in turn were important factors for childhood diarrhea.

Maternal occupation was found to be significantly associated with childhood diarrhea. The findings of this study suggested that children of mothers whose occupation was non-agriculture had a higher risk of diarrhea compared with children whose mothers were not working; this result is in line with a case control study in Iran [24]. A study in Egypt showed a result quite contrary to this; children whose mothers were not working or farmers or manual laborers had a significantly higher frequency of diarrhea [16]. In Nigeria, mothers in informal occupations had 23% more likelihood of child diarrhea compared to mothers in other occupational categories [14]. The effect of maternal occupation on childhood diarrhea varied from study to study; this might be due to the difference in economic activities of each place studies took place.

In this study, the number of underfive children and birth order were found to be significantly associated with childhood diarrhea. As the number of children and birth order increased, the frequency of diarrhea increased significantly, and this finding is consistent with other studies done in Ethiopia, Egypt, Eritrea, and Pakistan [8,16,25,26]. When the number of children in the household and child birth order increases it is expected that children will be more vulnerable to diarrhea mainly because the quality of care and attention from parents decreases.

The results of this study showed that the likelihood of diarrhea in the two week period reached its peak at 12–23 months of age and began to fall after 24 months of age. Children above 24 months of age were at very low risk of diarrhea. This pattern was consistent with studies done in Sub-Saharan countries [1]. In Ethiopia, for instance, the peak occurs among infants of 6–11 and 12–23 months of age [15]. In Nigeria the frequency of diarrhea was highest among children of 6–11 months of age, the period when most children start additional food [27]. Very young children 0–5 months of age were at a lower risk of diarrhea according to this study, and this clearly indicates that the protective effects of breastfeeding and less exposure of children to contaminated agents. On the other hand, the frequency of diarrhea peaks at the age 12–23 months when the child is exposed to different types of infections due to eating foods that are prepared un-hygienically, in unclean water and unhealthy environment. In this study, the age of a child showed a significant association with the occurrence of diarrhea which is in line with studies conducted in Uganda and Eritrea [21,25] that infants and children at 0–5 and above 24 months were at lower risk of diarrhea, and ages of 6–11 months and 12–23 months were at higher risk of diarrhea. The higher risks at these ages could be attributed to the fact that children at these age are either crawling or walking and can easily pick dirt or other contaminated objects for playing or eating.

This study clearly indicated that children of uneducated mothers were the most vulnerable to diarrhea in the absence of toilet facilities. This is consistent with a study in Ethiopia and Ghana [28,29]. Thus, educated mothers without toilet facilities can manage to reduce the risk of diarrhea even though these children are exogenously exposed to a higher risk of diarrhea. While children in households without toilet facilities were the most vulnerable, the risk was lower with mothers who had better education; this might indicate differences in parentally provided input, particularly on hygiene and nutrition. The findings also revealed considerably lower risks of diarrhea in children from a better educated mother with similar household facilities. Therefore, mothers’ education had a significant impact on diarrheal morbidity among children.

## Conclusion

This study has identified important socio-economic and environmental determinants that contribute to the occurrence of diarrhea in underfive children.

The independent variables that were found to be determinates were maternal education, maternal occupation, type of toilet facility, child’s stool disposal, and number of under five children, birth order, and age of child. A higher risk of diarrhoea was seen in children in a higher birth order and in households without improved toilet facilities. Lack of toilet facility was also a risk factor for childhood diarrhoea. When the analysis was stratified by maternal education, it became evident that children of uneducated mothers were the most vulnerable to diarrhoea in the absence of toilet facility. Mothers with some education protected their children against diarrhoea better than mothers with no education under poor environmental settings. There was an interaction effect of type of toilet facility with maternal education.

### Recommendations

In general, the findings of this study have important policy implications for health intervention programs and underline the view that encouraging girls’ education may have a considerable importance on child health and survival in the region. The long term solution for reducing the morbidity from diarrhea might involve the provision of better sanitation, and expanding family planning services so that they are accessible to the entire population.

Effective educational programs that emphasize hygiene should be strengthened in combination with health extension workers’ (HEW) educating households on sanitation through their house to house visits. Expanding family planning services to the entire population of the region will be helpful. Provision of continuous information improves the environmental conditions at household levels. Encouraging female school enrolment should be strengthened. A longitudinal study may be the best design to provide data in different seasons by including behavioural and other relevant factors.

### Strength and limitations of the study

#### Strength

In the absence of longitudinal and clinical data, cross-sectional surveys lend a hand for assessing the determinants and patterns of childhood diarrhea in the two weeks preceding the survey. Such findings may be of relevance for health intervention programs in the region.

#### Limitations

• We used a data that was collected cross-sectionally; in such studies it is difficult to take into account seasonal differences of the occurrences of diarrhoea.

• Diarrheal morbidity was measured by asking mothers about their childrens’ health in the past two weeks preceding the survey. This question measures mother’s perception of her child’s health instead of morbidity according to a clinical examination. Since perception of illness is not similar among different social groups, this may create variations across different socio-economic groups.

• Although some factors other than the ones we considered in this study affect diarrhoeal morbidity, we were not able to account for them. The most important one is breastfeeding practice because information on this variable was collected for children below 24 months of age.

## Competing interests

The authors declare that they have no competing interests.

## Authors’ contributions

TS designed the study, extracted the data, performed the analysis and interpretation of data and drafted the paper. GA assisted with the design, approved the proposal, and revised drafts of the paper. AS assisted with the design, approved the proposal, and revised drafts of the paper and prepared and revised the manuscript. All authors read and approved the final manuscript.

## Pre-publication history

The pre-publication history for this paper can be accessed here:

http://www.biomedcentral.com/1471-2431/14/102/prepub

## Supplementary Material

Additional file 1 Data extraction questionnaires. Click here for file
