# Supplementary material for: Determinants of childhood diarrhea among underfive children in Benishangul Gumuz Regional State, North West Ethiopia
Source: BMC Pediatr. 2014 Apr 14;14:102. doi: 10.1186/1471-2431-14-102 (PMC4021233; doi:10.1186/1471-2431-14-102)
Supplement: Additional file 1 — Data extraction questionnaires. [file 1471-2431-14-102-S1.docx]

**Data Extraction Questionnaire**

| **No** | **Questions** | **Answer** |
| --- | --- | --- |
| **Socio-economic characteristics** | | |
| 1 | Type of place of residence | 1. Urban 2. Rural |
| 2 | Mother’s highest educational level attainment | 1. No education 2. Primary 3. Secondary and above |
| 3 | Mother’s occupation | 1. Not working 2. Agriculture 3. Non agriculture |
| 4 | Father’s educational attainment | 1. No education 2. Primary education 3. Secondary and above |
| 5 | Ethnicity of mother | 1. Orthodox Christian 2. Muslim 3. Others |
| 6 | Religion of mother | 1. Gumuz 2. Berta 3. Amhara 4. Oromo 5. Others |
| 7 | Number of household members |  |
| 8 | Number of children 5 and under in the household |  |
| 9 | Type of floor material of the house | 1. Dirt 2. Non dirt |
| 10 | Wealth status of the household | 1. Poor 2. Medium 3. Rich |
|  | Environmental characteristics of the household |  |
| 11 | Source of drinking water | 1. Improved 2. Not improved |
| 12 | Type of toilet facility | 1. Improved 2. Not improved |
| 13 | How do you dispose the child’s stool | 1. Safe 2. Not safe |
| **Demographic and other factors** | | |
| 14 | Mother’s age |  |
| 15 | Childs age in months |  |
| 16 | What is the birth order number of a child |  |
| 17 | Size of a child at birth | 1. Larger than average 2. Average 3. Less than average 4. Very small |
| 18 | Sex of a child | 1. Male 2. female |
| 19 | Has a child had diarrhea in the last two weeks | 1. Yes 2. No |
